# Severe disseminated *Veillonella parvula* infection including endocarditis, bilateral psoas abscess, discitis, and osteomyelitis but sparing spinal and hip prostheses: a case report

**DOI:** 10.1186/s13256-022-03386-8

**Published:** 2022-04-20

**Authors:** Tobias Richards, Juan Stephen, Chok Lin Lui

**Affiliations:** Geriatric Medicine, St John of God Midland Public and Private Hospitals, 1 Clayton St, Midland, WA 6056 Australia

**Keywords:** Back pain, *Veillonella parvula*, Disseminated infection, Occult infection, Infective endocarditis, Psoas abscess, Osteomyelitis

## Abstract

**Background:**

*Veillonella* species are an opportunistically pathogenic commensal anaerobic Gram-negative coccus commonly found in the oral, genitourinary, respiratory, and intestinal tract of humans and some animals. Infection is rare, even in immunocompromised hosts, and has been identified to cause a wide array of different infections, including endocarditis, osteomyelitis, and meningitis.

**Case presentation:**

An 82-year-old Caucasian male retired ex-gymnast presented to the emergency department with a 2-week history of acute on chronic lower back pain without clear precipitant. He displayed no systemic symptoms, and had not sustained any recent injuries. Initial blood and radiological investigation did not reveal an infective or mechanical cause for his pain; however, a few days into admission, he developed a fever and signs of sepsis. A thorough septic screen was performed, including a spinal magnetic resonance imaging scan, which did not reveal any abnormalities. Blood cultures revealed *Veillonella parvula* bacteremia, with subsequently repeated magnetic resonance imaging displaying rapid disseminated infection including bilateral psoas abscess, discitis, and osteomyelitis. Infective endocarditis was later identified with echocardiogram. He received intravenous ceftriaxone and later oral amoxicillin and clavulanic and recovered on 6-month follow-up.

**Conclusions:**

This case illustrates the potential pathogenicity and unexpected rapid course of *Veillonella parvula* infection even in an immunocompetent host presenting with back pain. This case highlights the critical importance of a thorough septic screen when investigating patients for early signs of sepsis.

## Introduction

*Veillonella parvula* is a commensal anaerobic Gram-negative coccus commonly found in the oral, genitourinary, respiratory, and intestinal tract of humans and some animals [1]. *Veillonella* species are a rare cause of serious infections, including meningitis, osteomyelitis, prosthetic joint infections, pleuropulmonary infection, endocarditis, and bacteremia [1–3]. *Veillonella* species produce an endotoxic lipopolysaccharide with capacity for opportunistic infections in immunocompromised hosts [4, 5]. While *Veillonella* species have been identified as causing a wide array of different infections, disseminated infection is rare especially in combination with spinal infection [6]. Three species of *Veillonella* have been reported to cause human infections, including *Veillonella atypica*, *Veillonella dispar*, and *Veillonella parvula* [7]*.* We present a rare case of a patient with disseminated *Veillonella parvula* infection that included evidence of bacteremia, infective endocarditis, bilateral psoas muscle abscesses, thoracic vertebral discitis, epidural phlegmon, and tibial plateau osteomyelitis without involvement of the patient’s bilateral hip prosthesis and spinal metallic fixation devices. This report is unique as it describes the pathogenic capacity of *Veillonella* species, and how this was investigated and successfully treated. This case is an important contribution to the developing knowledge of *Veillonella* species and will assist clinicians in the investigation and management of patients with identified infections with this pathogen.

## Case presentation

An 82-year-old Caucasian male retired ex-gymnast from home presented with a 2-week history of acute on chronic lower back pain without a clear precipitant. The pain was exacerbated when weight-bearing, causing the man to collapse to the floor in pain. There were no associated fevers, sweats, or neurological symptoms. The patient’s past medical history included spinal osteoarthritis with an L2–S1 decompression laminectomy and fusion with transpedicular screws and spinal rods last revised in 2008, C4–C5 decompressive laminectomy and anterior cervical discectomy and fusion in 1992–1994, and bilateral hip replacement in 2005. The patient’s pain was managed by his general practitioner, he was taking pregabalin 150 mg twice a day, buprenorphine patch 5 mg per hour weekly, and codeine 30 mg for pain flares. There was no history of recent dental procedures, infections, or other surgeries. There was no history of immunodeficiency or corticosteroid use. The patient was a lifelong nonsmoker and drank up to two standard drinks of alcohol occasionally on weekends.

On physical examination in the emergency department, his vital signs were unremarkable, with heart rate 90 beats per minute, blood pressure 135/70 mmHg, respiratory rate of 16 breaths per minute, oxygen saturation of 96% on room air, and temperature of 36.5 °C. On palpation, he was moderately tender generally at the L4 and L5 vertebrae, and unable to complete a straight leg raise owing to pain when raising his leg. There were no remarkable neurological, respiratory, or cardiac findings. On assessment in the emergency department, his full blood count (white cell count 9.0 × 10^9^/L), C-reactive protein (CRP; < 0.7 mg/L), and renal function blood tests were within normal limits and unremarkable. A computed tomography scan (CT) of the thoracolumbar spine demonstrated advanced degenerative changes. He was admitted under the geriatric team for analgesia and allied health assessment with a provisional diagnosis of acute on chronic flare in degenerative osteoarthritis. Two days into his admission, a septic screen was performed when he spiked a temperature of 38 °C and exhibited tachycardia of 112 beats per minute and hypotension of 90 mmHg systolic. His blood tests revealed a CRP of 279 mg/L with a white cell count of 9.2 × 10^9^/L, and the patient was commenced on empirical intravenous ceftriaxone 2 g 24-hourly. An urgent lumbosacral MRI performed on the same day revealed no features of discitis or epidural abscesses. An abdominal portovenous CT scan was performed to investigate for a possible gastrointestinal source, as well as pelvic and femoral X-rays of the hip prosthesis looking for evidence of osteomyelitis, but all were unremarkable. All four blood cultures taken during the septic screen revealed *Veillonella* species, which was later confirmed to be *Veillonella parvula*.

**Fig. 1:**
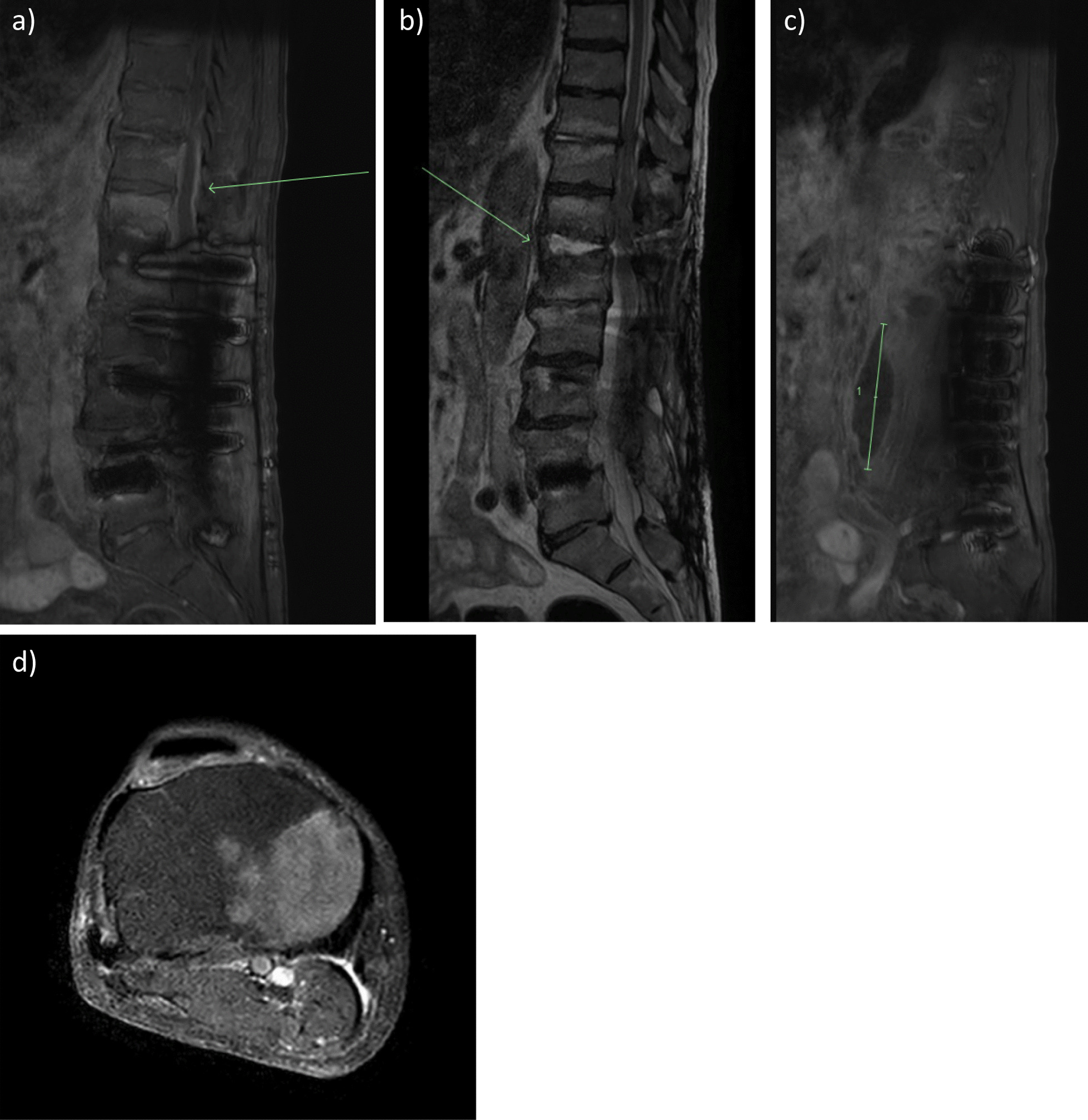
(**a**) Arrow is showing epidural enhancement and thickening consistent with T10–L1 epidural phlegmon. No discrete walled-off epidural abscess is present. (**b**) Arrow is showing Discitis at T12–L1, there is marrow oedema and enhancement within the T12 and L1 vertebral bodies and the T12/L1 intervertebral disc is oedematous with endplate erosions. Changes are in keeping with T12/L1 discitis and adjacent vertebral body osteomyelitis. (**c**) Arrow is pointing to the right psoas abscess extending from L1 to L4 measuring 2.4 cm (AP) × 2.4 cm (transverse) × 8.3 cm (craniocaudal). This extends from L1 to L4. Oedema is seen within the psoas muscles surrounding the collection. (**d**) Right knee tibial plateau osteomyelitis demonstrated by marrow oedema and enhancement involving the medial aspect of the tibial plateau and proximal tibial metaphysis

An infectious disease consultation was requested following the initial MRI, and the antibiotic regimen was changed to intravenous piperacillin/tazobactam 4.5 g 8-hourly. In addition to the lower back pain, the patient was also developing new right knee pain. On examination, there was general joint tenderness and reduced range of movement due to pain; although it was unclear if this pain was due to the patient’s known osteoarthritis or underlying infective cause. To investigate for disseminated infection, a whole-body bone scan single-photon emission computerized tomography was performed 4 days later and demonstrated evidence of T12–L1 discitis and features of osteomyelitis in the right tibial plateau. Repeat lumbosacral and right knee MRI 5 days following first MRI demonstrated a T10–L1 effacing epidural phlegmon, T10–T12 discitis, bilateral psoas abscesses (the largest extending from L1–L4), and right tibial plateau osteomyelitis (Fig. 1). Transthoracic echo identified small vegetations on the mitral and tricuspid valves. Further investigation with transesophageal echo was not performed as the patient was deemed unsuitable for cardiothoracic intervention owing to his age. His right psoas abscess was drained percutaneously, and microscopy, culture, and sensitivity of the abscess aspirate did not demonstrate significant bacterial growth. Sensitivities to the blood cultured *Veillonella parvula* confirmed sensitivity to amoxicillin/clavulanic acid, ceftriaxone, clindamycin, and metronidazole but resistance to penicillin. The patient was recommenced on intravenous ceftriaxone daily, leading to improvement in his back and knee symptoms. He was discharged on oral amoxicillin and clavulanic acid 875 mg/125 mg 12-hourly for 6 months. The primary source of this patient’s infection remains unclear. On follow-up, the patient made a full recovery after 6 months, and returned to his baseline level of function and mobility without evidence of infection reoccurrence.

## Discussion

We report herein a case of an immunocompetent man with acute on chronic back pain that took a unique course resulting in fever and the diagnosis of unexpected and rapidly progressive and disseminated *Veillonella parvula* bacteremia. This case is unique owing to the extensive spread of infection, as well as sparing of any joint or prosthesis involvement in the infection, despite spine and joint involvement and growth on blood cultures obtained from the patient.

*Veillonella* species have been identified in the literature to be a rare cause of infection in humans. The majority of these cases have host susceptibility factors such as being in the extremes of age, having had recent surgery or trauma, or having immunosuppression [1]. To the best of the authors’ knowledge, this is the first case report to document such severely disseminated *Veillonella parvula* infection, as well as such infection without predominant susceptibility factors being present [6]. Also, while evidence exists for *Veillonella* species having a predilection for prosthesis, it is remarkable that this patient was spared any involvement in his bilateral hemiarthroplasty or vertebral stabilization rods from vertebral fusion surgery [8–11].

In our patient, there was no obvious risk factor for immunodeficiency apart from age, and he was otherwise independent at his place of residence. It is unclear if age alone was a sufficient factor to predispose to infection; there were no known sources identified on examination or history of this patient such as recent surgery or endoscopic procedures. Prior case reports have identified a diagnostic delay of up to 2 months in patients presenting with infective spondylodiscitis, with most patients having some degree of immunosuppression [6]. It is remarkable that our patient developed such a diffuse infection, which highlights the potential pathogenicity of the *Veillonella* species.

Of note in this case report is the speed and rapid progression of infection. It is remarkable that the initial workup for this patient failed to demonstrate any evidence of infection with normal laboratory investigations when he presented with a 2-week history of progressive severe lower back pain. Once fever had become apparent, investigation with lumbosacral MRI also failed to identify any evidence of spinal infection, which had become apparent later in the course. Within 5 days, the patient demonstrated radiological progression of disease, including bilateral psoas abscess, discitis, and epidural phlegmon and osteomyelitis, suggesting rapid progression of the infection even with broad-spectrum antibiotics. Timely blood cultures taken at the onset of fever before initiation of antibiotic therapy were crucial to the detection of this patient’s infection and guiding treatment. All four blood culture sets taken identified the same organism and allowed for directed antibiotic therapy.

After *Veillonella parvula* infection is confirmed on microbiological sample testing, the mainstay of treatment is intravenous antibiotics, which can be further specified after antibiotic sensitivity has been completed. In the literature, there is an absence of clear consensus or guidelines on the treatment of *Veillonella parvula* infections owing to the limited reports available [8]. Available case reports documenting the treatment of *Veillonella parvula* infection most commonly report the effective use of intravenous cephalosporin antibiotics such as ceftriaxone or in combination such as cefotaxime and metronidazole for a 6-week period [13–16]. These antibiotics are then either ceased if there is complete improvement, or switched to oral penicillin antibiotic such as amoxicillin or amoxicillin/clavulanic acid and oral metronidazole for a further 4 weeks [13, 15]. One study identified a *Veillonella parvula* infection sensitive to chlorphenicol, and was successfully treated with a 2-week course of intravenous course of chlorphenicol when there was incomplete response to intravenous meropenem [17]. Patients who respond incompletely to antibiotics or develop abscesses may require surgery and drainage to fully treat infection, although this is uncommon in the literature as most infections respond well to antibiotic therapy [13].

## Conclusion

In conclusion, this case illustrates the potential pathogenicity and unexpected course of *Veillonella parvula* infection in an immunocompetent patient presenting with acute on chronic back pain. This case highlights the importance of approaching lower back pain with a broad range of differential diagnoses and care in the investigation of patients with unexpected fever. This case cautions clinicians managing patients with *Veillonella parvula* bacteremia to be aware of its virulent capacity, and the importance of performing thorough and timely septic investigations.

## Data Availability

All data and material for this case were sourced from the hospital unit and electronic medical records.
